# Experimental Investigation on Laser Impact Welding of Fe-Based Amorphous Alloys to Crystalline Copper

**DOI:** 10.3390/ma10050523

**Published:** 2017-05-12

**Authors:** Xiao Wang, Yapeng Luo, Tao Huang, Huixia Liu

**Affiliations:** School of Mechanical Engineering, Jiangsu University, Zhenjiang 212013, China; yapengluo@126.com (Y.L.); huangtao102510@163.com (T.H.); lhx@ujs.edu.cn (H.L.)

**Keywords:** laser impact welding, Fe-based amorphous alloys, surface wave, wavy interface

## Abstract

Recently, amorphous alloys have attracted many researchers’ attention for amorphous structures and excellent properties. However, the welding of amorphous alloys to traditional metals in the microscale is not easy to realize in the process with amorphous structures unchanged, which restrains the application in industry. In this paper, a new method of welding Fe-based amorphous alloys (GB1K101) to crystalline copper by laser impact welding (LIW) is investigated. A series of experiments was conducted under different laser energies, during which Fe-based amorphous alloys and crystalline copper were welded successfully by LIW. In addition, the microstructure and mechanical properties of welding joints were observed and measured, respectively. The results showed that the surface wave and springback were observed on the flyer plate after LIW. The welding interface was straight or wavy due to different plastic deformation under different laser energies. The welding interface was directly bonded tightly without visible defects. No visible element diffusion and intermetallic phases were found in the welding interface. The Fe-based amorphous alloys retained amorphous structures after LIW under the laser energy of 835 mJ. The nanoindentation hardness across the welding interface showed an increase on both sides of the welding interface. The results of the lap shearing test showed that the fracture position was on the side of copper coil.

## 1. Introduction

With the continuous development of the manufacturing industry, more attention has been paid to performance requirements of materials to meet further complex production requirements. In addition, most products need to adapt to a variety of severe environments, such as corrosion resistance and high temperature creep resistance. Therefore, single layer metal materials cannot meet the needs of modern industrial development [1]. Thus, the joining of dissimilar materials is becoming an inevitable trend in industrial applications due to their advantages. Amorphous alloys have shown a great potential for their amorphous structures and excellent mechanical, magnetic, high strength and corrosion resistance properties [2,3]. Copper has attracted a lot of attention because of its excellent thermal conductivity and electrical conductivity. Thus, the joining of amorphous alloys with copper can combine the excellent properties of two materials, which will have wide engineering application prospects in industries [4]. However, the welding methods of dissimilar materials such as selective laser melting [5,6,7], fusion welding, brazing welding, [8,9] and laser joining [10] are not suitable for the welding of amorphous alloys. Because much heat and intermetallic compounds are easily produced in the welding interface during these welding processes, which can destroy the amorphous structures and weaken the welding strength of the welding joints.

In order to make amorphous alloys more useful and extend their industrial application fields, it is necessary to investigate a method to realize the welding of amorphous alloys to other materials. Recently, the welding of amorphous alloys to other materials has been investigated by solid state welding processes such as explosive welding (EXW), friction stir welding (FSW) and vaporizing foil actuator welding (VFAW) processes [11]. Chiba et al. [2,12] achieved the welding of bulk metallic glass (BMG) to traditional metal plates by EXW, and the wavy interface was observed in the welding interface. They also found there was no devitrification occurred in the bonding interfaces by X-ray diffraction. In addition, they found the amorphous structure of the BMG was unchanged and the mechanical properties of the BMG still maintained the original properties. Sun et al. [13] studied friction stir spot welding of the thick Zr_55_Cu_30_Al_10_Ni_5_ BMG to pure Cu plates, whose welding interfaces were clean. In addition, no obvious crystallization was found from the amorphous structure. Sun et al. [14] also investigated the friction stir butt welding of Zr_55_Cu_30_Al_10_Ni_5_ bulk metallic glass (BMG) to pure copper plates. The result of mechanical test showed that the hardness of the base metals was higher than the welding joint. Moreover, it was found that the fracture position of the tensile test was on the copper side. Li et al. [15] successfully achieved the welding of Zr_46_Cu_46_A_l8_ BMG to crystalline pure Al without any defects by using FSW, and there was no crystallization and reaction layer detected in the welding interface between the BMG and Al. Vivek et al. [16] concluded that VFAW was a feasible means to weld Zr-based BMG to copper. The result showed that the grain refinement of the welding interface resulted in the increase of the hardness in the copper sheets. Furthermore, no crystallization was found on the BMG throughout the welding process. In these above methods, the excellent bonding between traditional metals and BMG can be achieved without obvious crystallization in the BMG. However, it is not applicable for some smaller thinner micro-device welding with the above-mentioned several kinds of welding methods.

In recent years, laser impact welding (LIW) has attracted many researchers’ interest and the experimental principle was similar to general impact-driven welding. LIW was the best for micro-device welding of similar and dissimilar metals [17]. Daehn and Lippold [18] successfully obtained the welding combinations of two metals by LIW at a low temperature. Zhang et al. [17] then studied the welding of similar and dissimilar metals with three different welding means. The wavy interface was observed and the micro-hardness along the interface was improved. Additionally, compared with the other two kinds of welding methods, intermetallics were not observed after LIW. Wang et al. [19] then studied the LIW of similar and dissimilar metals, which were welded successfully, and they concluded that LIW was a feasible method to weld metals with a small size employing the proper impact angle and laser energy. Wang et al. [20] studied the effect of ablative layers and connection methods on the height of the dimple formed on the flyer of LIW. Wang et al. [21] also successfully achieved the welding of similar and dissimilar metal plates by LIW, and they found that the interface was flat and wavy. With the change of impact angle, the wavelength and amplitude of the interface wave changed continuously along the welding interface. They concluded the same results by simulation.

Due to the fact that amorphous alloys and traditional metals are not easy to be directly welded in the microscale, a new method of welding Fe-based amorphous alloys (GB1K101) to crystalline copper by laser impact welding (LIW) was investigated. The purpose of the current study was to weld Fe-based amorphous alloys (GB1K101) to crystalline Cu under four different laser energies with LIW. The morphology of the welding samples was observed by optical microscopy (OM); the welding interfaces under different laser energies were observed through metallographic investigation by scanning electron microscopy (SEM); and the structure of amorphous alloys after LIW was measured and discussed. In addition, the nanoindentation hardness and the bonding strength of joints were also tested.

## 2. Mechanism of Laser Impact Welding

**Figure 1:**
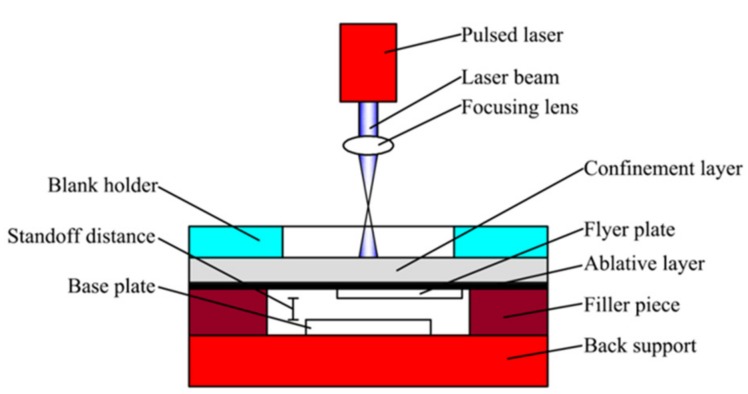
Schematic diagram for laser impact welding.

The basic experimental setup of LIW is shown in Figure 1, which is mainly composed of back support, filler piece, base plate, flyer plate, ablative layer, confinement layer and a blank holder. K9 glass with a thickness of 3 mm is served as the confinement layer due to its good light transmission property and impact strength. A thin black lacquer layer is used as the ablative layer, which is painted on K9 glass. The flyer plate is tightly stuck to the ablative layer by using the cyanoacrylate adhesive (Loctite 380). The base plate paired with the flyer plate is placed on the back support. The standoff distance can be changed by adjusting the thickness of the filler piece. In addition, the whole experimental setup is compressed tightly with a blank holder to restrain the leakage of plasma induced by laser pulse [22].

**Figure 2:**
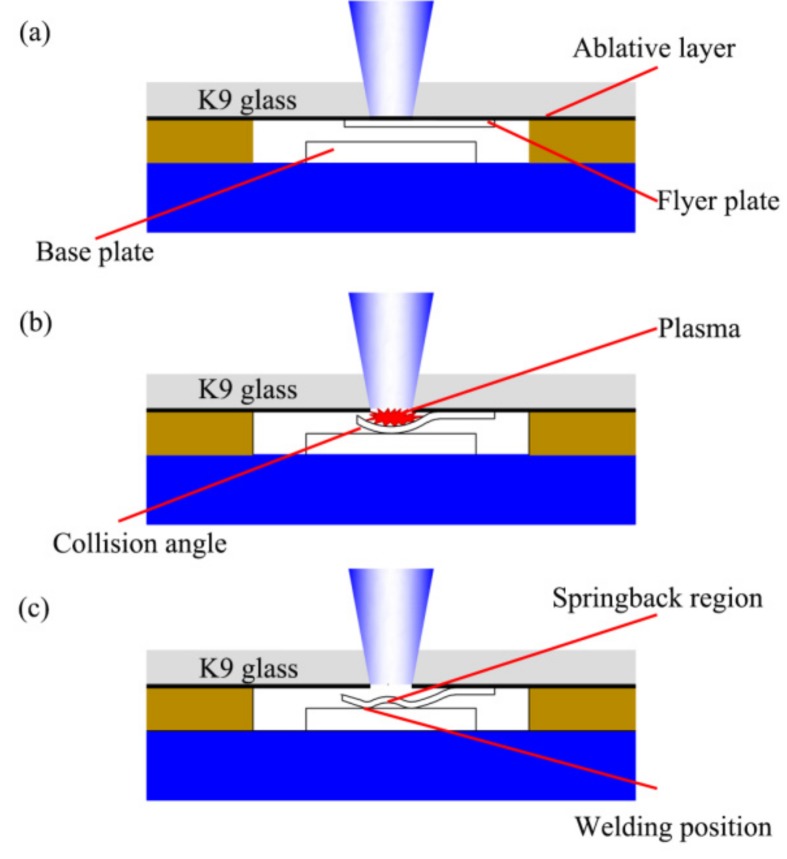
Experimental principle of laser impact welding: (**a**) the black lacquer absorbs laser energy and vaporizes; (**b**) the flyer collides onto the base plate at a high speed; (**c**) after welding.

The basic experimental process of the LIW is shown in Figure 2. First, the laser beam irradiates the ablative layer through the blank holder and confinement layer (K9 glass). Under the irradiation of the laser beam, the black lacquer absorbs laser energy and vaporizes instantaneously [23], as shown in Figure 2a. Then, the vapor continues absorbing laser energy and ionizing into a high-temperature and high-pressure plasma. The plasma is restricted by the confinement layer and the blank holder; thus, high pressure created by plasma propagates into the flyer plate in the form of the shockwave [24,25]. The shockwave expands downwards and drives the flyer plate towards the base plate at a high speed. In addition, this process is accomplished in several microseconds [17], as shown in Figure 2b. During this process, the collision angle varies from zero to some optimum values. At the beginning of collision, the center of the plates cannot be bonded due to insufficient collision angle and speed. When the collision angle and speed reach a certain value, the jet will produce between the flyer plate and base plate. Then, the surface oxide layer on the flyer plate and the base plate will be removed by the jet so as to form the fresh surface for welding [21]. Otherwise, the metallurgical bonding could not be obtained. Once the impact speed exceeds a certain value, the springback will occur on the flyer plate, as shown in Figure 2c.

## 3. Experimental Materials and Equipment

In this study, pure copper (T2) and Fe-based amorphous alloys (GB1K101) were welded by LIW with thicknesses of 0.03 mm and 0.028 mm, which were selected as flyer plate and base plate, respectively. In order to obtain fine grain structure, the copper foils were annealed at 720 K for 2 h [21]. The chemical compositions of the Fe-based amorphous alloys (GB1K101) and copper foils are given in Table 1 and Table 2, respectively. Before welding, the materials for welding were cut into special dimensions, and the dimensions of the flyer plate and base plates were 20 mm × 5 mm and 20 mm × 20 mm, respectively.

In the experiment, the short pulse Nd:YAG laser beam with Gaussian distribution was used and its main parameters are shown in Table 3. The proper laser spot size (1.5 mm) is achieved by adjusting the distance between the working platform and the table focusing lens. The energy of the laser pulse used in this experiment is 835, 1200, 1550, and 1800 mJ, respectively. After the LIW, the black lacquer remaining on the upper surface of welding spots was removed with anhydrous alcohol. In order to observe the welding interface and conduct hardness tests conveniently, the welding samples were cut close to the welding spot from samples first. Then, the welding samples were grounded by sandpaper with a roughness from #80 to #3000. Finally, they were polished by diamond paste of 1.0 μm and 0.5 μm. A KEYENCE VHX-1000C optical microscope (KEYENCE Corporation, Osaka, Japan) with depth-of-field and high resolution was used to observe the surface morphology and the welding interface of samples. The glassy phase of amorphous alloys after LIW was tested by X-ray diffractometry using CuKα radiation (XRD, D8 ADVANCE, Bruker, Karlsruhe, Germany). The wavelength used in X-ray diffraction is 1.15418 Å. A scanning electron microscopy (SEM, Hitachi Corporation, Tokyo, Japan) equipped with an energy dispersive spectroscopy (EDS, EDAX Corporation, Mahwah, NJ, USA) was used to observe the microstructure of the Cu/amorphous alloys (GB1K101) welding interface. To evaluate the micro-hardness of the welding interface, the welding interface was measured by a NanoIndenter CSM (Anton Paar, Graz, Austria), with a Berkovich diamond indenter, whose maximum load is 10 mN. Lap shearing tests were conducted by using an Instron Type UTM 4104 testing machine (SUNS Corporation, Shenzhen, China), and the speed in the test was 1 mm/min. The lap shearing tests were conducted three times for different laser energies (835 mJ, 1200 mJ, 1550 mJ, and 1800 mJ pulse energy).

## 4. Results and Discussion

### 4.1. Weld Samples

**Figure 3:**
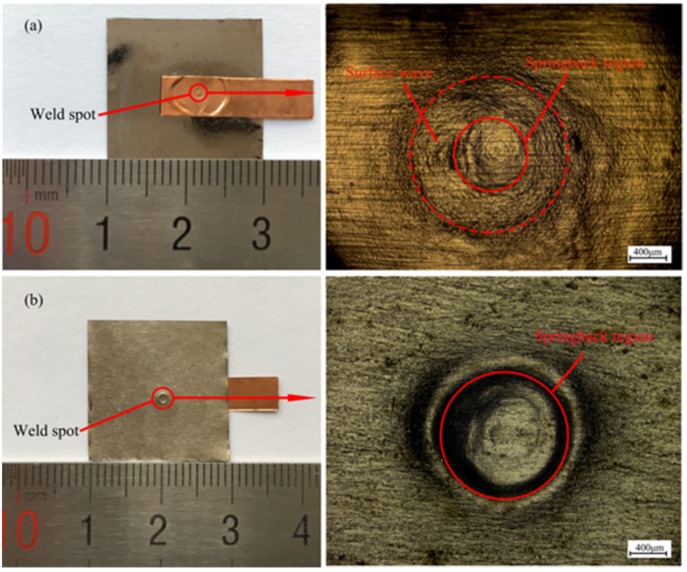
Weld sample and surface morphology of welded joints by LIW at 1500 mJ laser pulse energy: (**a**) upper surface of the joint; (**b**) bottom surface of the joint.

The experiment results showed that copper foils and Fe-based amorphous alloys (GB1K101) were successfully welded through using 1.5 mm laser spot diameter and 0.2 mm standoff distance under four different laser energies. Figure 3 shows the welding sample and surface morphology of joints produced at 1500 mJ pulse energy. As we can see in Figure 3a, a surface wave between the solid circle and the dashed circle was formed on the collision region of the flyer plate after the laser impact welding. This may be because when the shockwave expanded downwards and drove the flyer plate to collide with the base plate, high impact pressure would occur at the collision point. The pressure reached the GPa level, so the pressure wave was generated between welding materials, which propagated outwards. Series of reflections and refractions would occur when the pressure wave encountered the interface. Under the effect of reflection and refraction of the pressure wave, the flyer plate produced irreversible plastic deformation, thus forming the surface wave. The area of the surface wave on the flyer plate is annular, which is different from that of explosive welding. The area of the surface wave of explosive welding is formed along the explosion direction, while the area of the surface wave of laser impact welding is formed by moving outward from the center of the impact point along the radius.

Springback in the center of the flyer plate and the back of base plate was observed in Figure 3a,b, and the springback in the flyer plate was also found by other research [21,22]. The reason for the springback on the base plate can be explained as follows: when the shock wave drove the flyer plate to collide with the base plate at a high speed, plastic deformation occurred between the flyer plate and the base plate. Because the thickness of the base plate is thinner, the flyer and base plate continued to move and collide with the back support. Then, back support absorbed partial energy induced by the shockwave, which further led to the forming of the crater on the back support. At the same time, the other energy induced by the shockwave would act on the base plate by reaction force, resulting in the plastic deformation on the base plate. Thus, the springback on the base plate was formed.

### 4.2. Microstructure of the Welding Interface

**Figure 4:**
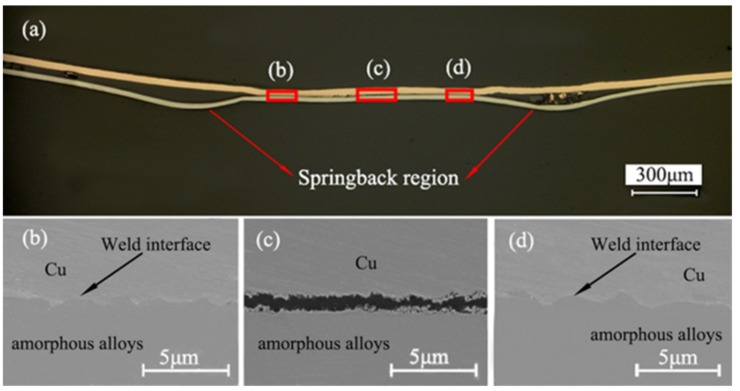
The welding interface of Cu/amorphous alloys joint produced at 1550 mJ pulse energy: (**a**) cross-section of the welding sample under the optical microscope; (**b**–**d**) magnified views of regions **b**–**d** marked in (**a**) under the SEM.

Figure 4a shows the optical micrograph of the polished cross-sectional area of the bonding interface of the welding sample under 1550 mJ energy. The springback area between the red arrows on the base plate is obvious, which corresponds to the springback area of Figure 3b. Figure 4 demonstrates that the interface of Cu/amorphous alloys is bonded with good quality. The excellent wavy interface is observed in Figure 4b,d, which further demonstrates that copper foils and amorphous alloys are successfully welded by LIW. The wavelength and amplitude is about 2.7 μm and 0.8 μm, respectively. The wavy interface was also found by Chiba et al. [2,12], but this welding is in the macroscale. However, the center of the welding spot is not welded, as shown in Figure 4c. Thus, the joint of laser impact welding was accomplished with an annular bonding region. The reason may be the effect of the collision angle and springback. The collision angle of the center region of the welding spot did not reach a certain value, so the welding interface of the center region was not obtained. The same phenomenon was found by other research [22,26]. They found when the collision angle reached a certain value, bonding took place between the flyer plate and the base plate and continued until the collision angle exceeded the threshold. In addition, the bonding interfaces were pulled apart due to the effect of the springback on the flyer plate. As we can see, the welding quality of Cu/amorphous alloys in the center is not good.

As we can see, the wave interface in Figure 4b,d is very obvious without visible defects, and the intermetallic phases is not found in the weld interface.

**Figure 5:**
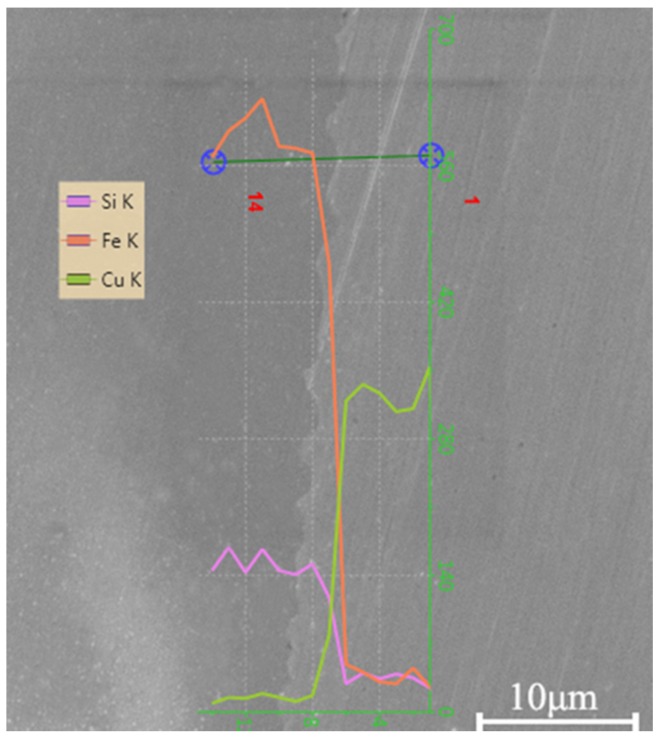
Elemental distribution in the welding interface for Cu/amorphous alloys.

Figure 5 shows the contents of different elements in the welding interface by using the EDS method. The curves indicate the contents of different main elements along the scanning line. The content of Fe and Si elements almost remains unchanged on the side of the amorphous alloys, while it decreases sharply in the welding interface. However, the content of the Cu element increased sharply from the amorphous alloys to copper in the welding interface. It shows that there is no element diffusion in the welding interface. The reason could be explained as follows: the peak pressure of the shock wave induced by the laser is up to the GPa level in the LIW process, but the welding time is only maintained for a few microseconds [17] and the temperature of the interface decreases sharply. Therefore, it is difficult to provide sufficient time and heat for element diffusion. In addition, no visible defects in the welding interface are formed, which restrains the diffusion of metal elements. Thus, there is no visible element diffusion. The same phenomenon was also found by Chiba et al. [2] and Liu et al. [22].

**Figure 6:**
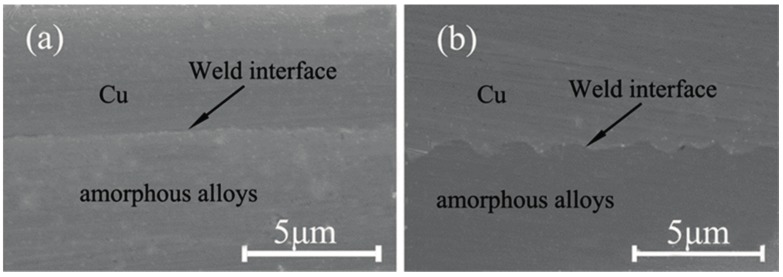
SEM micrographs of the welding interfaces obtained at different pulse energies: (**a**) 835 mJ; (**b**) 1550 mJ.

Figure 6 shows the different SEM micrographs of the welding interfaces at two different pulse energies. The effect of increasing energy on the welding interface wave can be observed clearly. Figure 6a shows that the interface micrograph is straight and a little wavy under the 835 mJ laser energy, while the interface micrograph is a smooth wave under the 1550 mJ laser energy in Figure 6b. This was because, when the loading energy was 835 mJ, the velocity on the collision point was low, and low shock velocity led to little shock pressure. Then, the plastic deformation produced in the welding interface was so small that the interface was straight. However, when the loading energy was 1500 mJ, a higher shock velocity would be produced on the collision point, and then the high shock velocity created high plastic deformation and shear stress in the welding interface where the smooth wave interface was formed. Some previous research [27,28,29] concluded the same results. They found that straight and wavy interfaces can also be obtained in explosive welding, and an increase in explosive loading increased the impact energy of the flyer plate. This caused the transition from a straight interface to a wavy interface [27]. Similar results were also concluded by other researchers, and they thought that the welding interface was straight due to the fact that the explosive loading was insufficient for obtaining a wavy interface [30]. Compared with the explosive welding, the wave of LIW is relatively small. This is because the energy in LIW is lower than that in explosive welding. Moreover, the materials used in LIW are thinner than that in explosive welding.

**Figure 7:**
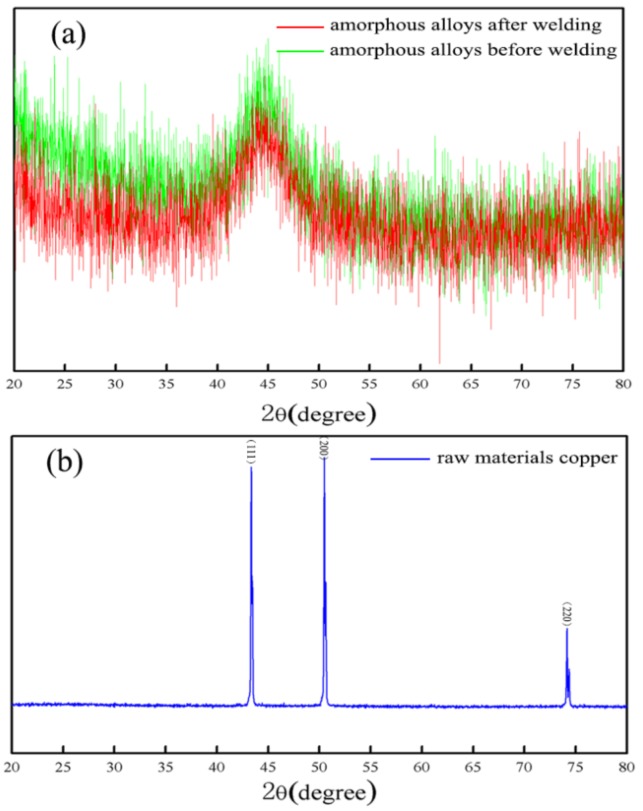
Micro-focused X-ray diffraction patterns of the welding sample produced at 835 mJ: (**a**) amorphous alloys before and after welding; (**b**) raw material copper.

XRD analysis was performed on the welding sample produced at 835 mJ. The green line in Figure 7a shows the X-ray diffraction pattern from raw amorphous alloys (GB1K101) at room temperature. As we can see, in the whole range of scanning angles, only a slight change in the intensity of the scattered X-ray is observed, and one diffraction peak that is not sharp is seen clearly. No detectable amounts of other phases are observed, which is the typical diffraction pattern of amorphous materials. The blue line in Figure 7b shows the X-ray diffraction pattern from the crystalline Cu plate at the room temperature, and it has three obvious diffraction peaks. The peaks are sharp and independent. XRD were carried out on the amorphous alloy side of the welding samples produced at the 835 mJ, in order to confirm whether the amorphous materials after LIW were due to crystallization or not. From the red line in Figure 7a, we can determine the X-ray diffraction pattern from the amorphous alloys side of the welding samples after LIW is similar to that of raw amorphous alloys. The above results reveal that the amorphous alloys (GB1K101) keep amorphous structures after LIW when the laser energy was 835 mJ, and no crystallization occurred in the welding process. Some previous research [14,15] confirmed the same results. The reason for this phenomenon may be that the welding process of LIW was accomplished within several microseconds, and the temperature in the welding interface dropped down rapidly to the temperature below the crystallization temperature, which did not provide enough heat to cause devitrification in the welding process. Therefore, the amorphous structure was not destroyed, and the amorphous alloys still retained excellent properties under the 835 mJ laser energy.

### 4.3. Mechanical Properties

#### 4.3.1. Nanoindentation Hardness Test

**Figure 8:**
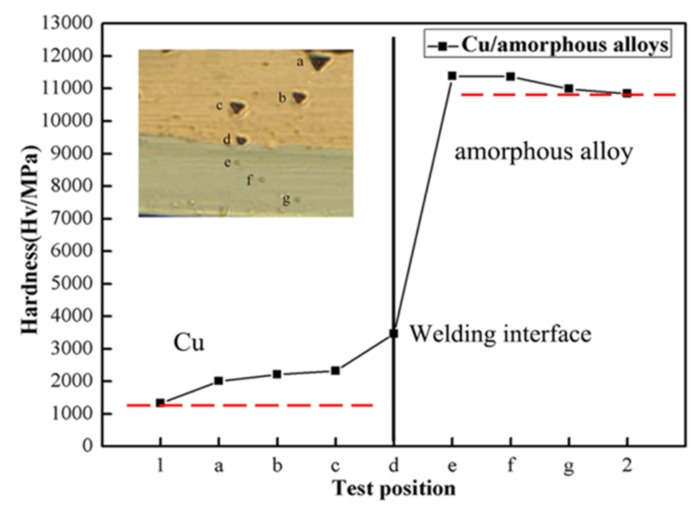
Nanoindentation hardness distribution across the interfaces.

A nanoindentation hardness test was carried out on both sides of the interface of the welding sample produced at 1550 mJ pulse energy. During the test, 10 mN loads were applied to the welding interface. Figure 8 shows the hardness distribution in the welding interface, where the position d of the horizontal ordinate represents the welding interface. Positions 1 and 2 show the hardness of raw materials, respectively. The test positions are shown in the inset of Figure 8. The original hardness of copper and amorphous alloys was also tested with the hardness of 1324.6 Mpa and 10,841 Mpa, respectively. The hardness of Cu near the welding interface (position c) is 2324.7 Mpa, which improved a lot compared to the raw materials. The similar conclusion can be concluded on the side of amorphous alloys. As shown in Figure 8, the indentation depth of copper is deeper than that of amorphous alloys due to its low hardness. The nanoindentation hardness in the welding region (position d) is higher than copper but lower than amorphous alloys. With the position of the test from outside the welding interface, the nanoindentation hardness of the flyer and base plate increases gradually. A similar result was also found by Liu et al. [31] and Wang et al. [21]. The phenomenon may be that the high plastic deformation occurred in the welding interface, which led to grain refinement of the welding interface and further work hardening. However, the hardness on the side of the copper increases remarkably compared with an amorphous alloy. This was due to the fact that laser impact welding is a high strain welding process and the plastic deformation that occurred on the flyer plate and base plate was different. The self-hardness of the amorphous alloy is so high, and the plastic deformation of amorphous alloy is small, so the increase of hardness on the side of amorphous alloys is slow.

#### 4.3.2. Lap Shearing Test

**Figure 9:**
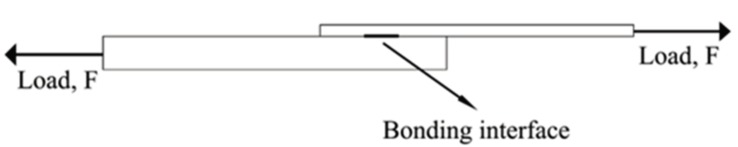
Illustration of lap shearing test.

**Figure 10:**
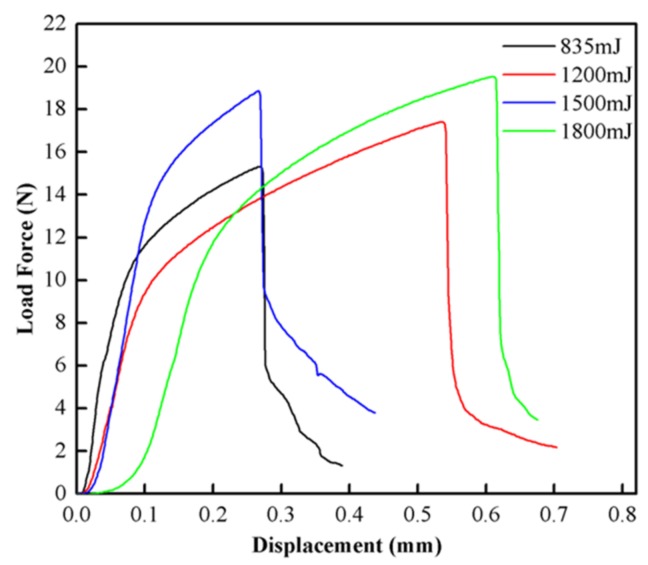
The force–time curves with different laser energies.

**Figure 11:**
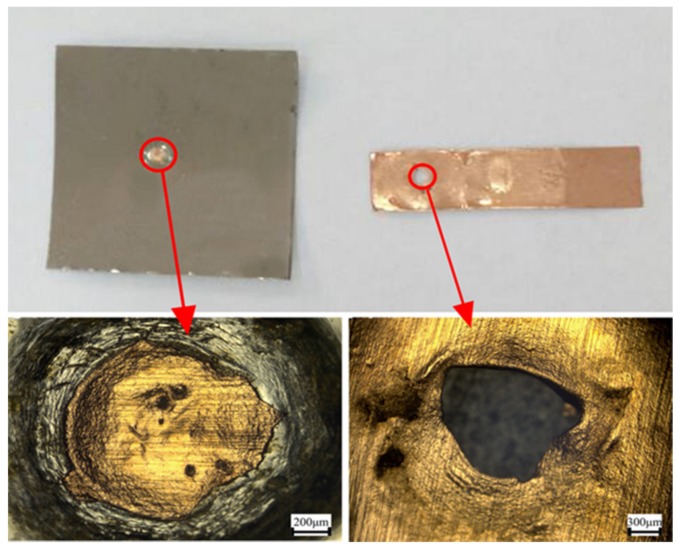
Failure modes of the welding sample under 1500 mJ energy.

A lap shearing test is an important means to measure the welding strength of welding samples. In order to measure the welding strength, lap shearing tests were performed on the welding samples of LIW under different energies. The welding samples under different energies were tested three times to obtain the average value. Figure 9 shows the test setup. Figure 10 shows the lap shearing tests curves for the Cu/amorphous alloys welded joint. It is clear that the average failure loads of welding samples are 15.32, 17.42, 18.87 and 19.54 N under different energies, respectively (835 mJ, 1200 mJ, 1550 mJ and 1800 mJ pulse energies). In addition, with the increase of laser energy, the failure load of the welding joints increases continuously. This can be explained as follows: the plastic deformation became larger when the laser energy increased, and the welding interface varied from a straight to wavy interface. Then, the mechanical interlocking formed between the wavy interfaces, which led to improvement of the welding strength of the welding sample [22]. In addition, the wavy interface contributed to the increase in the welding area, so the friction between the welding interfaces increased, which further increased the maximum failure loads. It can be seen that the trend of the force–time curves with different energies is the same. From the curve, we can know that the maximum load force decreases suddenly when the maximum load force reaches a specific value, and the fracture of the welding sample may occur suddenly at this time. Figure 11 demonstrates that the fracture position is on the side of copper coils. It shows that the welding strength of Cu/amorphous alloys is greater than that of Cu foils, which demonstrates that the welding interface is reliable. The same result was concluded by other research [14,16]. The shape of the copper left on amorphous alloys is a circle, which corresponds to the annular welding region in Figure 3.

## 5. Conclusions

In this paper, the copper (T2) foils and Fe-based amorphous alloys (GB1K101) were successfully welded with better welding quality by LIW. The welding interface was observed by optical microscopy (OM) and scanning electron microscopy (SEM), and the mechanical properties of the welding samples were carried out by nanoindentation hardness and a lap shearing test. The main conclusions drawn from the article can be summarized as follows:

1. The copper (T2) foils and Fe-based amorphous alloys (GB1K101) were successfully welded by LIW. The surface wave was observed on the flyer plate and the springback was also found on the flyer plate and base plate.
2. Straight and wave interfaces were observed in the welding interface due to different extents of the plastic deformation. The welding interfaces were directly bonded without intermetallic phases and visible element diffusion.
3. The Fe-based amorphous alloys (GB1K101) retain amorphous structures after LIW under the 835 mJ laser energy. The nanoindentation hardness of the welding interface was obviously improved after LIW.
4. The failure load of the welding joints increased with the increase of laser energy and the fracture position of welding samples was on the side of copper coils.

## Figures and Tables

**Table 1:** Chemical composition of 1K101 (weight %).

Elements	Fe	Si	B
1K101	78~80	8~10	12~14

**Table 2:** Chemical composition of copper (weight %).

Elements	Cu + Ag	Bi	Sb	As	Fe	Pb	S	Other
Copper	99.9	0.001	0.002	0.002	0.005	0.005	0.005	0.01

**Table 3:** Main parameters of Spitlight 2000 Nd:YAG Laser.

Parameters	Values
Pulse energy	80–1800 mJ
Pulse width	8 ns
Wave length	1064 nm
Energy stability	<±1%
Exit spot diameter	9 mm
